# Patterns of Immediate-Release and Extended-Release Opioid Analgesic Use in the Management of Chronic Pain, 2003-2014

**DOI:** 10.1001/jamanetworkopen.2018.0216

**Published:** 2018-06-01

**Authors:** Catherine S. Hwang, Elizabeth M. Kang, Yulan Ding, Josephine Ocran-Appiah, Jana K. McAninch, Judy A. Staffa, Cynthia J. Kornegay, Tamra E. Meyer

**Affiliations:** 1 University of Washington School of Medicine, Seattle; 2 Office of Surveillance and Epidemiology, Center for Drug Evaluation and Research, Division of Epidemiology, US Food and Drug Administration, Silver Spring, Maryland; 3 Now with Chiltern International Inc, King of Prussia, Pennsylvania; 4 Now with GlaxoSmithKline, Rockville, Maryland

## Abstract

**Importance:**

Many stakeholders are working to improve the safe use of immediate-release (IR) and extended-release/long-acting (ER/LA) opioid analgesics. However, little information exists regarding the relative use of these 2 formulations in chronic pain management.

**Objectives:**

To describe the distribution of IR and ER/LA opioid analgesic therapy duration and examine adding and switching patterns among patients receiving long-term IR opioid analgesic therapy, defined as at least 90 consecutive days of IR formulation use.

**Design, Setting, and Participants:**

A retrospective cohort study of 169 million individuals receiving opioid analgesics from across 90% of outpatient retail pharmacies in the United States from January 1, 2003, to December 31, 2014, using the IQVIA Health Vector One: Data Extract Tool. Analyses were conducted from March 2015 to June 2017.

**Exposures:**

Receipt of dispensed IR or ER/LA opioid analgesic prescription.

**Main Outcomes and Measures:**

Distribution of therapy frequency and duration of IR and ER/LA opioid analgesic use, and annual proportions of patients receiving long-term IR opioid analgesic therapy who added an ER/LA formulation while continuing to use an IR formulation, switched to an ER/LA formulation, or continued receiving IR opioid analgesic therapy only.

**Results:**

Among the 169 280 456 patients included in this analysis, 168 315 458 patients filled IR formulations and 10 216 570 patients filled ER/LA formulations. A similar percentage of women received ER/LA (55%) and IR (56%) formulations, although those receiving ER/LA formulations (72%) were more likely to be aged 45 years or older compared with those receiving IR formulations (46%). The longest opioid analgesic episode duration was 90 days or longer for 11 563 089 patients (7%) filling IR formulations and 3 103 777 patients (30%) filling ER/LA formulations. The median episode duration was 5 days (interquartile range, 3-10 days) for patients using IR formulations and 30 days (interquartile range, 21-74 days) for patients using ER/LA formulations. From January 1, 2003, to December 31, 2014, a small and decreasing proportion of patients with long-term IR opioid analgesic therapy added (3.8% in 2003 to 1.8% in 2014) or switched to (1.0% in 2003 to 0.5% in 2014) an ER/LA formulation.

**Conclusions and Relevance:**

Most patients receiving opioid analgesics, whether for short or extended periods, use IR formulations. Once receiving long-term IR opioid analgesic therapy, patients are unlikely to add or switch to an ER/LA formulation.

## Key Points

**Question:**

What percentage of patients receiving long-term immediate-release (IR) opioid analgesic therapy in the United States adds or switches to an extended-release/long-acting (ER/LA) formulation?

**Findings:**

In this cohort study, most patients (96.0%) receiving IR formulations for 90 days or longer continued IR opioid analgesic use without adding (3.3%) or switching (0.7%) to an ER/LA product. Furthermore, many patients received only 1 IR (40%) and/or 1 ER/LA (41%) prescription throughout the 12-year study period.

**Meaning:**

Most patients receiving opioid analgesics use IR formulations, and patients receiving long-term IR opioid analgesic therapy are unlikely to add or switch to an ER/LA formulation.

## Teaser

This cohort study characterizes use of immediate-release and extended-release/long-acting opioid analgesic therapy formulations and adding and switching patterns between the 2 formulations among patients in the United States between 2003 and 2014.

## Introduction

Growing awareness of opioid misuse, abuse, and overdose has prompted stakeholders to reconsider prescribing practices for opioid analgesics. Past teaching that encouraged health care professionals to aggressively treat both acute pain and chronic pain with opioid analgesics is being challenged, and clinical guidelines are being reexamined.^1,2,3,4^ Recently, many professional societies, states, and medical systems have recommended or required limited prescribing of extended-release/long-acting (ER/LA) formulations for both acute pain and chronic pain.^5^ Additionally, the Centers for Disease Control and Prevention recommends that clinicians initially prescribe immediate-release (IR) formulations rather than ER/LA formulations for chronic pain.^6^ However, little information exists regarding the frequency with which patients add or switch to ER/LA opioid analgesic therapy after long-term IR opioid analgesic use.

Although prior investigations have examined patterns of opioid analgesic use, many only assessed specific products or multiple products at an aggregate level.^7,8,9,10,11^ Other studies were limited by their geographic scope, cross-sectional designs, or small sample sizes.^12,13^ Given numerous recent efforts to address the opioid crisis, including the US president’s declaration of a public health emergency^14,15^ and the report by the President’s Commission on Combating Drug Addiction and the Opioid Crisis,^16^ there is a growing urgency to better understand national drug use patterns for IR and ER/LA formulations. Therefore, using a national source of longitudinal prescription dispensing data, we examined the annual proportion of patients using IR formulations for 90 days or longer who added an ER/LA formulation while continuing the use of an IR formulation, switched to an ER/LA formulation, or continued receiving IR opioid analgesic therapy only.

## Methods

### Data Source

In this cohort study, we used the IQVIA Vector One: Data Extract Tool (DET) to follow individual patients longitudinally from January 1, 2003, to December 31, 2014. The study follows the Strengthening the Reporting of Observational Studies in Epidemiology (STROBE) reporting guideline. The DET integrates prescription activity from various sources, including national retail chains, mass merchandisers, food stores, and provider groups. Varying amounts of data are captured from across 90% of pharmacies in the United States, representing approximately 56% of all prescriptions dispensed in the US outpatient retail setting. The database contains cash, Medicaid, and other commercial health insurance payer types, including Medicare Part D. Each prescription record is linked to a unique deidentified patient number so that individuals can be tracked longitudinally across multiple pharmacies and payers. The patient identifier algorithm uses a combination of variables available in prescription claims, such as first initial, last name, date of birth, and zip code. The algorithm is robust to changes in 1 or 2 aspects of the patient identifier, but multiple changes may result in a false new patient identifier. Our study was exempted from review by the US Food and Drug Administration (FDA) Research Involving Human Subjects Committee. Patient consent was not required because the data are deidentified. We conducted our analyses from March 2015 to June 2017.

### Cohort Derivation for Patient-Level Analyses

We searched Uniform System of Classification^17^ codes and product names to identify IR and ER/LA opioid analgesic prescriptions dispensed in the United States from January 1, 2002, to December 31, 2014 (eTable in the Supplement). We restricted our study to oral solid dosage forms and transdermal patches to avoid capturing cough syrups and products not indicated for pain. We excluded opium tincture, because its primary indication is for diarrhea, and buprenorphine, because it is often used for treating opioid use disorder. We also excluded patients with 1 or more prescription records that met the following criteria: unknown age; unknown sex; prescription refill number greater than 0 for schedule II controlled substances; prescription refill number greater than 5 for schedule III and IV controlled substances; days’ supply less than 1 or greater than 90 days; or quantity dispensed less than 1 or greater than 1000 units. These exclusion criteria were based on Controlled Substance Act regulations and judgment that the records likely did not represent valid prescriptions.^18^

### Statistical Analysis

We constructed episodes of continuous therapy for IR opioid analgesic prescriptions dispensed from January 1, 2003, to December 31, 2014. Immediate-release opioid analgesic prescriptions starting within 7 days of the end date of the previous IR opioid analgesic prescription were considered part of the same therapy episode. If 2 IR opioid analgesic prescriptions overlapped and there was no prescription following the second prescription, up to a maximum of 7 days was added to the end of the second prescription. Adding overlap days to the end of the second prescription did not delay the start date of the third prescription if another overlap period was present between the second and third prescriptions. This process was repeated to create ER/LA opioid analgesic episodes. A therapy episode may have included multiple active moieties.

Next, we conducted a retrospective analysis to examine annual opioid analgesic use patterns among patients receiving long-term IR opioid analgesic therapy, defined as having an IR analgesic episode lasting 90 days or longer. We excluded patients who filled an ER/LA opioid analgesic prescription within 180 days before the start of their first long-term IR opioid analgesic episode because we tried to avoid situations in which a patient’s recent use of ER/LA opioid analgesics may influence his or her adding and switching behavior.

We determined the annual proportion of long-term IR opioid analgesic users who added an ER/LA formulation while continuing to use an IR formulation, switched to an ER/LA formulation, or continued receiving IR opioid analgesic therapy only. We defined add-on therapy as filling 1 or more ER/LA opioid analgesic prescriptions during or within 30 days following the IR opioid analgesic episode and filling a new IR opioid analgesic prescription after the initiation of ER/LA opioid analgesic therapy. Therefore, this group included patients who continued to receive IR formulations after beginning ER/LA opioid analgesic therapy. We defined a switch as filling 1 or more ER/LA opioid analgesic prescriptions during the IR opioid analgesic episode or within a 30-day period after the end of the IR opioid analgesic episode, without filling a new IR opioid analgesic prescription before the end of the ER/LA opioid analgesic episode. Thus, these patients included only those who transitioned from IR opioid analgesic use to exclusive ER/LA opioid analgesic use. We defined continued IR opioid analgesic use as receiving no ER/LA opioid analgesic prescriptions between the beginning of the 180-day look-back period and 30 days after the end of the IR opioid analgesic therapy episode. Patients with more than 1 long-term IR opioid analgesic episode during the study period were included only once based on the start date of their first long-term IR opioid analgesic exposure. For the primary patient-level analysis, we excluded ER/LA opioid analgesic episodes that began at the same time as a long-term IR opioid analgesic episode because there was no clear temporal pattern for assessing adding or switching behavior. We also excluded ER/LA opioid analgesic episodes that started after but ended before the termination of a long-term IR opioid analgesic episode because of challenges in our ability to define this pattern as a true or temporary add-on. In addition, because our definitions of adding and switching behaviors were based on filling a new IR opioid analgesic prescription before the end of the ER/LA opioid analgesic episode, ER/LA opioid analgesic episodes contained within the IR opioid analgesic episode may be inadvertently classified as a switch. Therefore, we excluded these episodes to avoid these situations.

We conducted multiple sensitivity analyses. First, we redefined long-term IR opioid analgesic therapy as at least 30, 60, 180, or 365 days of continuous use to determine whether adding and switching patterns differed for shorter and longer durations of IR opioid analgesic use. Second, we repeated the main analysis using therapy episodes that allowed for a 15-day gap and overlap period between prescriptions. Third, we used an alternative episode definition in which we added a maximum of 7 days to the end of each overlapping prescription and delayed the start dates of subsequent prescriptions to account for the time of overlap. Fourth, we repeated the main analysis, including ER/LA opioid analgesic episodes that started simultaneously or were contained within an IR opioid analgesic episode as add-on therapy.

We performed all analyses using SAS statistical software version 9.4 (SAS Institute Inc).

## Results

### Characteristics of Patients Receiving IR and ER/LA Opioid Analgesics

A total of 169 280 456 patients were included in the final DET analytic sample, representing 94% of the original opioid analgesic data set after applying the exclusion criteria (Table 1). A total of 168 315 458 patients filled a total of 1 095 653 540 IR opioid analgesic prescriptions, and 10 216 570 patients filled 101 587 259 ER/LA opioid analgesic prescriptions from January 1, 2003, to December 31, 2014. A similar percentage of women received ER/LA (55%) and IR (56%) formulations. Seventy-two percent of patients receiving ER/LA formulations were aged 45 years or older, while 46% of patients receiving IR formulations were aged 45 years or older. Compared with IR formulations (28%), a greater proportion of ER/LA opioid analgesic prescriptions (35%) were paid for by Medicaid.

**Table 1.** Description of the Analytic Sample, 2003 to 2014^a^

Characteristic	No. (%)		
	Patients Receiving ER/LA Opioid Analgesics	Patients Receiving IR Opioid Analgesics	Patients Receiving Long-term IR Opioid Analgesic Therapy With No Prior ER/LA Opioid Analgesic Prescription^b^
Patients, No.	10 216 570^c^	168 315 458^c^	9 853 311^c^
Female	5 654 921 (55)	94 533 078 (56)	5 804 452 (59)
Age at first prescription, y			
0-17	98 931 (1)	12 855 772 (8)	106 149 (1)
18-44	2 752 085 (27)	78 193 663 (46)	3 586 569 (36)
45-64	4 375 663 (43)	51 337 961 (31)	4 070 612 (41)
≥65	2 989 891 (29)	25 928 062 (15)	2 089 981 (21)
Prescriptions, No.	101 587 259	1 095 653 540	427 518 953
Payer type			
Cash	8 462 042 (8)	140 645 393 (13)	49 923 214 (12)
Medicaid	35 858 231 (35)	306 871 650 (28)	148 167 694 (35)
Other commercial health insurance	57 266 986 (56)	648 136 497 (59)	229 428 045 (54)
Census region^d^			
Northeast	18 663 557 (18)	166 251 004 (15)	61 612 821 (14)
Midwest	21 797 763 (21)	241 035 048 (22)	93 403 093 (22)
South	35 576 225 (35)	427 723 800 (39)	177 183 225 (42)
West	25 403 500 (25)	255 316 500 (23)	94 141 713 (22)

Abbreviations: ER/LA, extended-release/long-acting; IR, immediate-release.

^a^ Source: IQVIA Vector One: Data Extract Tool, 2003-2014.

^b^ Includes patients with at least 1 episode of 90 days or longer of continuous IR opioid analgesic exposure and no ER/LA opioid analgesic prescription in the 180 days prior to their long-term IR opioid analgesic exposure.

^c^ Patient counts are not mutually exclusive because a patient may have received both an IR and ER/LA opioid analgesic prescription during the study period. A total of 169 280 456 patients were included in the analytic sample.

^d^ The analytic data set includes some missing data for census region.

### Duration of Use Analysis

Among patients with at least 1 IR or ER/LA opioid analgesic episode of any duration, a median of 2 (interquartile range [IQR], 1-8) prescriptions were dispensed (Table 2). A total of 67 715 645 patients (40%) receiving IR formulations and 4 169 566 patients (41%) receiving ER/LA formulations received only 1 prescription. In addition, 74 838 123 patients (44%) receiving IR formulations and 5 831 515 patients (57%) receiving ER/LA formulations had only 1 opioid analgesic therapy episode of any duration during the 12-year study period. Regarding long-term opioid analgesic use, 11 563 089 patients (7%) receiving IR formulations and 3 103 777 patients (30%) receiving ER/LA formulations had opioid analgesic episode durations of at least 90 days. The longest therapy episode lasted a median of 6 days (interquartile range [IQR], 4-17 days) for patients using IR formulations and 30 days (IQR, 27-120 days) for patients using ER/LA formulations. The median episode duration was 5 days (IQR, 3-10 days) for patients using IR formulations and 30 days (IQR, 21-74 days) for patients using ER/LA formulations.

**Table 2.** Duration of Use Analysis, 2003 to 2014^a^^,^^b^

Outcome	Patients Receiving ER/LA Opioid Analgesics	Patients Receiving IR Opioid Analgesics	Patients Receiving Long-term IR Opioid Analgesic Therapy With No Prior ER/LA Opioid Analgesic Prescription^c^
Patients, No.	10 216 570	168 315 458	9 853 311
Patients with ≥1 episode ≥90 d, No. (%)	3 103 777 (30)	11 563 089 (7)	9 853 311 (100)
Opioid analgesic prescriptions dispensed			
Prescriptions dispensed/patient, No.			
Mean (SD)	10 (20)	7 (16)	43 (39)
Median (IQR)	2 (1-8)	2 (1-5)	32 (17-58)
Patients with only 1 prescription, No. (%)	4 169 566 (41)	67 715 645 (40)	32 579 (0.3)
Opioid analgesic episodes			
Episodes/patient, No.			
Mean (SD)	3 (4)	4 (6)	14 (13)
Median (IQR)	1 (1-3)	2 (1-4)	11 (5-19)
Patients with only 1 episode, No. (%)	5 831 515 (57)	74 838 123 (44)	437 025 (4)
Duration of episodes			
Duration of episodes/patient, d			
Mean (SD)	76 (140)	14 (42)	90 (125)
Median (IQR)	30 (21-74)	5 (3-10)	55 (34-98)
Patients whose mean episode duration was ≥90 d, No. (%)	2 148 423 (21)	3 481 958 (2)	2 848 400 (29)
Longest episode			
Longest episode/patient, d			
Mean (SD)	140 (276)	35 (127)	337 (340)
Median (IQR)	30 (27-120)	6 (4-17)	209 (125-407)
Patients whose longest episode duration was ≥90 d, No. (%)	3 103 777 (30)	11 563 089 (7)	9 853 311 (100)

Abbreviations: ER/LA, extended-release/long-acting; IQR, interquartile range; IR, immediate-release.

^a^ Source: IQVIA Vector One: Data Extract Tool, 2003-2014.

^b^ The patients receiving ER/LA opioid analgesics column is limited to ER/LA opioid analgesic prescriptions only. The patients receiving IR opioid analgesics and patients receiving long-term IR opioid analgesic therapy (≥90 days) with no prior ER/LA opioid analgesic prescription columns are limited to IR opioid analgesic prescriptions only. Patients may have received both IR and ER/LA opioid analgesic prescriptions during the study period. A total of 169 280 456 patients were included in the analytic sample.

^c^ This cohort includes patients with at least 1 episode of 90 days or longer of continuous IR opioid analgesic exposure and no ER/LA opioid analgesic prescription in the 180 days prior to their long-term IR opioid analgesic exposure. All IR opioid analgesic prescriptions and episodes during the study period among this cohort of patients receiving long-term IR opioid analgesics are described. This may include shorter IR opioid analgesic episodes that occurred before or after a patient’s first long-term IR opioid analgesic episode.

### Adding and Switching Patterns Among Patients Receiving Long-term IR Opioid Analgesic Therapy

Ninety-six percent of patients with long-term IR opioid analgesic therapy continued receiving IR formulations until the end of their therapy episode without transitioning to an ER/LA formulation (Table 3). Among patients who began long-term IR opioid analgesic therapy in 2003, 3.8% of patients added an ER/LA formulation to their IR therapy and 1.0% switched to an ER/LA formulation (Table 4). Over the study period, the proportion of patients adding or switching to ER/LA formulations decreased, reaching 1.8% in 2014 for add-on scenarios and 0.5% for switching scenarios. Patients adding or switching to an ER/LA formulation used IR formulations for a mean (SD) of 173 (218) days and 221 (224) days, respectively, before introducing an ER/LA formulation to their therapy regimen.

**Table 3.** Overall Proportion of Long-term IR Opioid Analgesic Users Adding, Switching, and Continuing Opioid Analgesic Therapy and Time to First ER/LA Opioid Analgesic Prescription, 2003 to 2014^a^

Outcome	Patients With IR Opioid Analgesic Episodes ≥90 d, No. (%)^b^	Time to First ER/LA Opioid Analgesic Prescription, d	
		Mean (SD)	Median (IQR)
Added ER/LA formulation	328 742 (3.3)	173 (218)	113 (56-202)
Switched to ER/LA formulation	69 832 (0.7)	221 (224)	146 (105-243)
Continued receiving IR opioid analgesic therapy only	9 454 737 (96.0)		

Abbreviations: ER/LA, extended-release/long-acting; IQR, interquartile range; IR, immediate-release.

^a^ Source: IQVIA Vector One: Data Extract Tool, 2003-2014.

^b^ This cohort includes patients with at least 1 episode of 90 days or longer of continuous IR opioid analgesic exposure and no ER/LA opioid analgesic prescription in the 180 days prior to their long-term IR opioid analgesic exposure. Patients with more than 1 long-term IR opioid analgesic exposure during the study period are only included once based on their first long-term IR opioid analgesic exposure.

**Table 4.** Annual Adding and Switching Patterns Among Patients Receiving Long-term IR Opioid Analgesic Therapy for 90 Days or Longer, 2003 to 2014^a^^,^^b^

Outcome	% by Year											
	2003	2004	2005	2006	2007	2008	2009	2010	2011	2012	2013	2014
Added ER/LA formulation	3.8	3.8	3.8	4.0	3.8	3.6	4.0	3.4	3.0	2.5	2.0	1.8
Switched to ER/LA formulation	1.0	0.9	0.9	0.9	0.8	0.7	0.7	0.6	0.6	0.5	0.5	0.5
Continued receiving IR opioid analgesic therapy only	95.2	95.3	95.3	95.1	95.4	95.7	95.3	96.0	96.5	97.0	97.5	97.7

Abbreviations: ER/LA, extended-release/long-acting; IR, immediate-release.

^a^ Source: IQVIA Vector One: Data Extract Tool, 2003-2014.

^b^ Patients were classified as adding an ER/LA formulation while continuing to use an IR formulation, switching to an ER/LA formulation, or continuing to receive IR opioid analgesic therapy only during a given year if their first long-term IR opioid analgesic episode of 90 days or longer began during that year.

The sensitivity analyses were consistent with the main analyses. Adding or switching to an ER/LA formulation continued to be infrequent among long-term IR opioid analgesic users when long-term use was redefined as 30, 60, 180, or 365 days, when gaps allowed between prescriptions were increased to 15 days, and when longer episode durations were allowed using an alternative method for constructing therapy episodes. For these sensitivity analyses, the overall proportion of patients that added an ER/LA formulation ranged from 3.9% to 4.3%, and the overall proportion that switched to an ER/LA formulation was consistently 0.7% for the entire study period. The proportion of patients who added an ER/LA formulation to their long-term IR therapy increased to 9.7% when we included ER/LA opioid analgesic episodes that began simultaneously or were contained within long-term IR opioid analgesic episodes as add-on therapy.

## Discussion

We found that most individuals receiving opioid analgesics obtained only 1 or 2 short-duration prescriptions between 2003 and 2014. Long-term opioid analgesic use usually consisted of IR formulations only, with approximately 4 times as many patients receiving long-term IR therapy compared with long-term ER/LA therapy. Among patients receiving long-term IR opioid analgesic therapy, a small and decreasing proportion of patients added or switched to an ER/LA formulation, even after more than a year of continuous IR therapy.

Many policy makers, health care payers, and other stakeholders have begun implementing strategies to encourage or require health care professionals to prescribe limited supplies of medication when treating acute pain with opioid analgesics.^5^ Our data reveal that most patients received only a few short-duration prescriptions, even before recent widespread efforts were implemented to limit treatment duration. Nevertheless, efforts to improve proper disposal of unused medication after treatment are of continued importance because many patients report excess opioid analgesic supplies that are stored in unsecure locations,^19^ providing opportunities for later misuse, abuse, and diversion.

Our results suggest that the long-term use of IR formulations, generally without the addition of ER/LA formulations, remains the most common practice for chronic pain management with opioid analgesics. Although the reasons for patients’ and health care professionals’ preferences for IR formulations could not be examined in this study, IR formulations may be continued long-term because of their more flexible dosing schedules, availabilities in low-dose combinations with nonopioid analgesics, lower costs, and more favorable insurance reimbursement policies. Additionally, past regulatory actions^20,21,22,23,24^ and interventions^20,25,26,27^ have primarily emphasized the risks associated with ER/LA formulations, particularly for long-term use in patients with chronic pain, which may have preferentially raised awareness about the risks associated with ER/LA formulations. For example, in 2012 and 2013, the FDA required manufacturers of ER/LA opioid analgesics to implement a risk evaluation and mitigation strategy,^20^ fulfill postmarketing requirements,^21,22,23,24^ and strengthen warnings on product labels regarding abuse, misuse, addiction, overdose, and death. Together, these interventions sought to clarify ER/LA opioid analgesics’ appropriate indications for use and ensure that health care professionals and patients were aware of the risks associated with these medications, including the risk of addiction and overdose.^21,22,23,24^

The small and decreasing percentage of patients receiving long-term IR opioid analgesic therapy who added or switched to an ER/LA formulation is noteworthy, given the approval of multiple new ER/LA products in recent years. Patient convenience and improved pain control are reasons often cited by pharmaceutical manufacturers for seeking FDA approval of new ER/LA opioid analgesic products. The belief is that patients using IR opioid analgesics on a long-term basis may be transitioned to an ER/LA formulation to maintain more consistent drug levels for pain requiring around-the-clock treatment. However, our results suggest that this practice is rare and decreasing over time. In addition to regulatory actions, such as the ER/LA Opioid Analgesic Risk Evaluation and Mitigation Strategy program, many factors may have disproportionately affected the use of ER/LA formulations, including higher costs of many ER/LA opioid analgesic products, tiered formularies, and prior authorization programs. For example, 18 months after the implementation of a prior authorization program in 2012, Massachusetts Blue Cross Blue Shield noted a 50% decrease in claims for ER/LA formulations, but only a 20% decrease in claims for IR formulations.^27^ The steady decrease in the proportion of patients who added an ER/LA formulation after long-term IR therapy beginning in 2009 may be explained by growing numbers of efforts to address the opioid crisis. For example, the FDA approved reformulated abuse-deterrent oxycontin in April 2010, an increasing number of states implemented prescription drug monitoring programs, more clinical guidelines cautioned physicians about the use of opioid analgesics for the treatment of nonmalignant pain, and many insurance companies implemented prior authorizations for prescribing opioid analgesics.^5^

Our results support the many efforts federal agencies^6,28,29^ have undertaken in recent years to enhance the safety and appropriateness of prescribing IR opioid analgesics. The reclassification of hydrocodone combination products from schedule III to schedule II in October 2014,^30^ labeling changes for IR opioid analgesics in March 2016,^31^ and expansion of the ER/LA Opioid Analgesic Risk Evaluation and Mitigation Strategy program to include IR formulations in September 2017^32,33^ are examples of regulatory actions designed to more effectively communicate the serious risks associated with IR formulations and to refine their indications of use. It will be important to monitor the short- and long-term effects of these actions to guide future interventions.

### Limitations

Our study has limitations. First, we lacked information about the clinical contexts and reasons for prescribing IR or ER/LA formulations. Second, we were unable to incorporate information about opioid analgesic dose in this study. Third, the data represented prescriptions dispensed rather than actual patient use. Fourth, the results of the adding and switching analysis were not nationally projected, and pharmacies that contributed data to the DET varied over the study period. However, the DET database contained a varying number of prescriptions dispensed from across 90% of all outpatient retail pharmacies in the United States. Although these analyses did not include a stable cohort over time, the large size of the database likely reflected general trends in opioid analgesic use in the US outpatient retail setting. Fifth, some patients in the study cohort may have received prescriptions from emergency departments, hospitals, or pharmacies not captured in the DET. While this is a limitation of all longitudinal analyses using prescription dispensing data, this study focused on trends in adding and switching behavior over time, and the proportions of uncaptured IR and ER/LA transactions were likely similar from one year to the next. Sixth, we did not consider the possibility of temporary adding and switching behavior because we did not examine future prescriptions for patients after an adding or switching event was identified. Seventh, to maximize use of the available data, we considered adding and switching patterns through the end of December 2014 and ignored the lack of a 30-day look-forward period for IR therapy episodes ending in December 2014. Sensitivity analyses that incorporated sufficient look-forward periods did not meaningfully change results.

Lack of insurance enrollment information prevented us from restricting the study population to patients who were continuously enrolled throughout the look-back period and study period, and the lack of a closed system prevented us from using an incident user design to examine IR to ER/LA adding and switching patterns. Nevertheless, our study may be more generalizable than other studies because while some claims databases allow investigators to account for continuous enrollment, they often only include patients who are commercially insured. In the DET database, approximately half of patients paid for their first opioid analgesic prescription using commercial health insurance, and nearly 50% of patients paid for their first opioid analgesic prescription using cash or Medicaid. Among patients who received long-term IR opioid analgesic therapy in our investigation, only 33% had the same payer type for all opioid analgesic prescriptions during the study period. Therefore, studies that exclusively use commercial claims data sources may miss a substantial proportion of opioid analgesic prescriptions, overall and within individual patients, contributing to a lack of generalizability and misclassification of prescription opioid analgesic exposures. Our study using DET data may be more generalizable to the population being prescribed opioid analgesics in the United States and may have less misclassification of continuous prescription opioid analgesic exposures.

Our findings stimulate further research questions. Recent articles have attempted to shed light on risk factors associated with long-term opioid analgesic use. For example, one study found that patients filling greater initial quantities, durations, and doses of opioid analgesics had higher risks of long-term opioid analgesic use.^34^ However, this investigation did not account for the intended treatment duration when the opioid analgesic was initially prescribed. Longer-term treatment may have been intended in patients initially prescribed greater amounts of opioid analgesics owing to increased disease severities or comorbidities, therefore confounding the observed correlation. Efforts to elucidate predictors for the development of opioid use disorder, as well as medically unnecessary transitions from acute to chronic opioid analgesic therapy, will help improve policies regarding appropriate opioid analgesic use.

Other areas of future work include examining adding and switching patterns among individual opioid analgesic active moieties and products. Furthermore, better understanding changes in prescribed IR and ER/LA opioid analgesic doses over time, reasons for choosing one formulation over another, and the generalizability of these findings to all settings of care will help guide opioid analgesic policies. As part of multipronged efforts to understand and address the current opioid crisis in the United States, a clear understanding of how IR and ER/LA formulations continue to be used in long-term pain management may help optimize the risk-benefit balance of these therapies, particularly as nonopioid therapies for chronic pain are developed and approved.

## Conclusions

Most patients receiving opioid analgesics, whether for short or extended periods, use IR formulations. In addition, once receiving long-term IR opioid analgesic therapy, patients are unlikely to add or switch to an ER/LA formulation, even after more than 1 year of continuous IR opioid analgesic therapy. Therefore, while recent FDA changes to IR opioid analgesic labeling may improve awareness of risks associated with IR formulations, ongoing efforts are needed to ensure their appropriate use in both acute pain and chronic pain settings.

## Notes

Supplement. **eTable.** Approved Extended-Release/Long-Acting Opioid Analgesic Products, 2003-2014
