# Supplementary material for: Patterns of Immediate-Release and Extended-Release Opioid Analgesic Use in the Management of Chronic Pain, 2003-2014
Source: JAMA Netw Open. 2018 Jun 1;1(2):e180216. doi: 10.1001/jamanetworkopen.2018.0216 (PMC6324408; doi:10.1001/jamanetworkopen.2018.0216)
Supplement: Supplement. — eTable. Approved Extended-Release/Long-Acting Opioid Analgesic Products, 2003-2014 [file jamanetwopen-1-e180216-s001.pdf]

Supplementary Online Content

Hwang CS, Kang EM, Ding Y, et al. Patterns of immediate-release and extended-release opioid analgesic use in the management of chronic pain, 2003-2014. *JAMA Netw Open*. 2018;1(2):e180216. doi:10.1001/jamanetworkopen.2018.0216

eTable. Approved Extended-Release/Long-Acting Opioid Analgesic Products, 2003-2014

This supplementary material has been provided by the authors to give readers additional information about their work.

© 2018 Hwang CS et al. *JAMA Network Open*.

eTable. Approved Extended-Release/Long-Acting Opioid Analgesic Products, 2003-2014[a]

Product	Active Moieties	Original Approval Date
Avinza®	Morphine sulfate	3/30/2002
Butrans®	Buprenorphine	6/30/2010
Dolophine®	Methadone HCl	8/13/1947
Duragesic®	Fentanyl	8/7/1990
Embeda®	Morphine sulfate and naltrexone	8/13/2009
Exalgo®	Hydromorphone HCl	3/1/2010
Kadian®	Morphine sulfate	7/3/1996
MethadoseTM	Methadone HCl	3/14/1973
MS Contin®	Morphine sulfate	5/29/1987
Nucynta® ER	Tapentadol HCl	8/25/2011
Opana® ER (new)	Oxymorphone HCl	12/9/2011
Opana® ER (old)	Oxymorphone HCl	6/22/2006
Oramorph SR	Morphine sulfate	8/15/1991
OxyContin®	Oxycodone HCl	12/12/1995
Palladone	Hydromorphone HCl	9/24/2004
Ryzolt	Tramadol HCl	12/30/2008
Targiniq® ER[b]	Oxycodone HCl, naloxone	7/23/2014
Ultram® ER	Tramadol HCl	9/8/2005
XartemisTM XR	Oxycodone HCl/acetaminophen	3/11/2014
Zohydro® ER	Hydrocodone bitartrate	10/25/2013

[a]Generic versions for some of these products were also available during the study period.
[b]This product was approved by the US Food and Drug Administration, but was not marketed.

© 2018 Hwang CS et al. *JAMA Network Open*.
